# Commonly Used Anesthesia/Euthanasia Methods for Brain Collection Differentially Impact MAPK Activity in Male and Female C57BL/6 Mice

**DOI:** 10.3389/fncel.2019.00096

**Published:** 2019-03-28

**Authors:** Mee Jung Ko, Grace E. Mulia, Richard M. van Rijn

**Affiliations:** ^1^Department of Medicinal Chemistry and Molecular Pharmacology, College of Pharmacy, Purdue University, West Lafayette, IN, United States; ^2^Purdue Institute for Integrative Neuroscience, Purdue University, West Lafayette, IN, United States; ^3^Purdue Interdisciplinary Life Sciences Graduate Program, Purdue University, West Lafayette, IN, United States

**Keywords:** ERK, JNK, p38, ketamine, isoflurane, carbon dioxide, central nervous system

## Abstract

The mitogen-activated protein kinases (MAPKs) are a family of protein kinases that regulate crucial neuronal functions such as neuronal differentiation, proliferation, and apoptosis through phosphorylation of subsequent protein kinases. The three classical MAPK subfamilies, extracellular signal-regulated kinase 1 and 2 (ERK1/2), c-Jun N-terminal kinase (JNK), and p38 kinase have been linked to various neurological disorders often in conjunction with activation of a wide range of G protein-coupled receptors and receptor tyrosine kinases. Many studies investigating MAPK function in these disorders rely on histochemistry or immunoblotting that require brain isolation following euthanasia. Here, we evaluated to what degree different modes of anesthesia/euthanasia impact MAPK activity in adult male and female C57BL/6 mice. Mice were decapitated following ketamine/xylazine or isoflurane anesthesia, carbon dioxide asphyxiation, or without anesthesia. We selectively chose five brain regions (the prefrontal cortex, the dorsal hippocampus, the dorsal striatum, the nucleus accumbens, and the amygdala) that are heavily implicated in neuropsychiatric disorders. We found that relative to carbon dioxide asphyxiation, the other methods displayed significantly stronger ERK1/2 phosphorylation in select brain regions of male and female mice, with no pronounced sex difference. A similar, yet, less pronounced trend was observed for JNK activity, whereas the choice of euthanasia method did not differentially impact p38 phosphorylation. Our study results reveal how small differences in experimental design may impact whether one will be able to detect drug- or disease-related changes in MAPK activity. These findings are timely in a period where experimental rigor is emphasized to increase reproducibility of research.

## Highlights

– Relative to carbon dioxide asphyxiation, isoflurane and decapitation significantly increased ERK1/2 activity in select mice brain regions.
– No significant sex difference of ERK1/2 activity was observed in tested brain regions.
– Compared to ERK1/2 activity, less pronounced trend was observed for JNK activity in all tested brain regions.
– Various euthanasia methods did not affect p38 activity in all tested brain regions.

## Introduction

The classical mitogen-activated protein kinases (MAPKs) comprise a family of three protein kinases, specifically extracellular signal-regulated kinases (MAPK1 and MAPK3), c-Jun N-terminal kinases (JNK, MAPK8–10), and p38 kinases (MAPK11–14). MAPKs have many functions including in the central nervous system (CNS) where they can regulate neuronal proliferation, differentiation, and apoptosis through phosphorylation and activation of subsequent protein kinases (Sweatt, 2001; Johnson and Lapadat, 2002). MAPKs are expressed in the soma, dendrites, and axons of neurons (Flood et al., 1998) as well as in glia and are integral in glioma formation and neurodegeneration (Stariha and Kim, 2001; Koistinaho and Koistinaho, 2002; Cheng et al., 2013). MAPKs can be phosphorylated following activation of receptor tyrosine kinases as well as G protein-coupled receptors, which can occur indirectly by cross-talk with receptor tyrosine kinases or more directly via protein kinase A and C signaling pathways (Marinissen and Gutkind, 2001; Wetzker and Böhmer, 2003; Kim and Choi, 2015). As part of the signaling transduction cascade of these receptors, MAPKs regulate behavioral performance such as fear conditioning and spatial learning (Besnard et al., 2014), and long-term synaptic plasticity in the brain (Thomas and Huganir, 2004), drug addiction (Lu et al., 2006), anxiety-like and depressive-like behavior (Huang and Lin, 2006; Duman et al., 2007; Wefers et al., 2012) signifying the importance of studying MAPK signaling pathways in various neuropsychiatric disorders. Interestingly, MAPKs have been found to scaffold with β-arrestin proteins (Shenoy and Lefkowitz, 2011) and linked for example to the aversive properties of κ-opioid receptor drugs (Bruchas and Chavkin, 2010; Ehrich et al., 2015).

Many studies investigating MAPK function in relation to neuropsychiatric disorders or drug efficacy rely on immunohistochemistry or immunoblotting that require brain extraction. However, the process of tissue collection may directly impact MAPK signaling and obscure any changes induced by a drug or disorder under investigation. Ketamine, which is often used in combination with xylazine to anesthetize animals prior to brain collection, is known to modulate MAPK signaling (Réus et al., 2014). Another commonly used agent for brain isolation is the anesthetic isoflurane, which may induce neuroinflammation and increase JNK phosphorylation (Altay et al., 2014). A third method, euthanasia by carbon dioxide asphyxiation, induces a hypoxic state and leads to activation of MAPK signal processes of cell survival (Risbud et al., 2005). Fourth, a rapid decapitation without anesthesia may trigger a stress response, and modulate MAPK signaling in the prefrontal cortex and the hippocampus (Meller et al., 2003).

Given the possibility of false negative results due to potentially high basal MAPK activity caused by the different modes of anesthesia/euthanasia, it is imperative to evaluate to what extent the choice of anesthesia/euthanasia can influence baseline MAPK activity. Thus, we assessed activation of extracellular signal-regulated kinase 1 and 2 (ERK1/2), JNK and p38 by Western blot using brain tissue collected from adult male and female C57BL/6 mice isolated following the four aforementioned anesthesia/euthanasia methods: ketamine/xylazine, carbon dioxide asphyxiation, isoflurane, and decapitation. For this investigation, we selectively isolated five brain regions, specifically the prefrontal cortex, the nucleus accumbens, the dorsal striatum, the dorsal hippocampus, and the amygdala, that are known for their roles in neuropsychiatric disorders. This is the first study to present the impact of different anesthesia/euthanasia methods in MAPK signaling pathway of the adult male and female mice brain. The findings provide crucial information to researchers who are seeking the optimal anesthesia/euthanasia method for their neuropsychological and pharmacological studies.

## Materials and Methods

### Animals

We utilized naïve C57BL/6 mice purchased from Envigo (Indianapolis, IN, USA). Young adult (7-week-old) male and female mice were group housed in ventilated Plexiglass cages (three mice per cage) on a reversed 12-h dark-light cycle (lights off at 10:00, lights on at 22:00, and used for experimental procedures when they reached 8–9-week-old (20 ± 2 g). Unless it is stated otherwise, three mice per group were used to test the molecular changes in the brain. Naïve mice groups were randomly assigned to each group. No animal was excluded from the study. All mice handling was performed by one scientist (MJK) to avoid stress induced by multiple handlers. Mice were maintained at ambient temperature (21°C) with *ad libitum* access to standard rodent diet and pathogen free reverse-osmosis water in an animal housing facility recognized by the Association for Assessment and Accreditation of Laboratory Animal Care. This study was carried out in accordance with the ARRIVE guidelines (Kilkenny et al., 2012) and the recommendations of the National Institutes of Health Guide for the Care and Use of Laboratory Animals. The protocol (#1305000864 by RR) was approved by the Purdue University Institutional Animal Care and Use Committee.

### Anesthesia/Euthanasia Methods

The mice brains were collected in a separate suite at the same time of the day during their active cycle following four different anesthesia and euthanasia methods: **(1) Ketamine/Xylazine**: mice were decapitated 45 min following an intraperitoneal injection of 100 mg/kg (#VINB-KET0-7021, Henry Schein Animal Health, Dublin, OH, USA) with 10 mg/kg xylazine hydrochloride supplement (#X1251-1G, Sigma-Aldrich, St. Louis, MO, USA). Serum ketamine levels are highest at 10–20 min post-injection (Ganguly et al., 2018); therefore to reduce the impact of ketamine on MAPK activity we chose the 45 min time-point, also because that is around the time a perfused brain would be collected. The anesthetic effect of a mixture of 100/10 mg/kg ketamine/xylazine is known to last up to 80 min with reflex suppressions and produces stable heart rates 40 min post-injection in mice (Erhardt et al., 1984; Xu et al., 2007); thus the mouse is still sedated at our chosen time point; **(2) Isoflurane**: mice were placed in a plexiglass chamber with 5% isoflurane, USP (#NDC 13985-046-60, VetOne, Boise, ID, USA) for 5 min, and decapitated when fully sedated, as measured by a lack of active paw reflex; **(3) Carbon Dioxide Asphyxiation**: mice were placed in a new cage with corn cob bedding, and immediately euthanized by displacement of air with 100% carbon dioxide, within 5 min and decapitated for tissue collection; and **(4) Decapitation**: mice were gently restrained and decapitated in a new cage to minimize the exposure to blood from conspecific mice.

### Tissue Collections and Sample Preparation

The mice brains were rapidly removed and coronal slices produced using a brain matrix (#RBMS-205C, Kent Scientific, Torrington, CT, USA). Sliced brains sections were flash-frozen with dry-iced chilled (−40°C) 2-methylbutane (#03551-4, Fisher Scientific, Waltham, MA, USA). The prefrontal cortex (Bregma = +2 mm to +5 mm), the dorsal hippocampus and the amygdala (Bregma = −2 mm to −1 mm), the dorsal striatum and the nucleus accumbens (Bregma = +0.5 mm to +1.5 mm) were collected using 1 mm biopsy punch (#15110-10, Miltex, Plainsboro, NJ, USA) based on the Paxinos and Franklin “the mouse brain in stereotaxic coordinates.” The designation of these regions is an oversimplification as the micro-punched tissue may also contain adjacent tissue or not fully encompass the entire region. Collected tissues were mixed with RIPA buffer and 1× protease inhibitor (#1861280, Fisher Scientific, Waltham, MA, USA) and homogenized with a Wheaton^®^ tissue grinder (#357535 and 357537, DWK Life Sciences, Millville, NJ, USA). Additional sample homogenization was performed using an ultrasonic disruption on ice (Level 3, 1-s per pulse, and 10 pulses total) using a probe-type sonicator (#XL-2000, Qsonica, Newtown, CT, USA), and centrifuged at 12,000 rpm, 4°C for 20 min. Supernatant of the samples were quantified using the BSA assay (#5000006, Biorad, Hercules, CA, USA) for further preparation for the Western blot. Samples were prepared with 4× Laemmli (#1610747, Biorad) and boiled at 98°C for 5 min prior to loading.

### SDS-Page and Western Blot

Twenty microliter samples containing 10 μg protein, as determined using a Bradford assay, were loaded in each well of a NuPage 4%–12% Bis-Tris gradient gel (#NP0336BOX, Fisher Scientific, Waltham, MA, USA) and transferred to nitrocellulose transfer membranes (#1620115, BioRad). The membrane was blocked in LiCor blocking buffer overnight at 4°C, and probed with the appropriate primary antibodies ERK1/2 (1:2,000, Cell Signaling 4696S, Lot: #22), pERK1/2 (1:2,000, Santa Cruz 7976-R, Lot: #C1113), p38 (1:2,000, Bioss 0637R, Lot: #AE020601), pp38 (1:500, Cell Signaling 9216S, Lot: #27), JNK (1:2,000, Cell Signaling 9252S, Lot: #17), pJNK (1:2,000, Santa Cruz 6254, Lot: #B2117), and α-Tubulin (1:2,000, Santa Cruz 5286, Lot: #G3117) for 1 h at room temperature. All membranes were washed in TBS based 0.1% Tween 20 solution (#9416, Sigma-Aldrich), and further probed with corresponding LiCor Near-infrared fluorescent secondary antibodies (1:5,000, LiCor 926-68020 Lot: #C60824-02, LiCor 926-32211 Lot: #C61103-06) for 2 h at room temperature. All membranes were washed in TBS based 0.1% Tween 20 solution (#9416, Sigma-Aldrich), and scanned using a LiCor Odyssey^®^ CLx Scanner. The use of LiCor secondary antibodies allowed us to detect the MAPK, pMAPK and α-Tubulin on the same blot without the need to strip the blots. Each band of the blots was cut based on their size (e.g., 42/44 kDa for ERK1/2 and 50 kDa for α-Tubulin). Because it is possible that the euthanasia method could impact MAPK expression, we also measured α-Tubulin as an internal loading control to ensure equal loading of samples.

### Statistical Analysis

All data are presented as means ± standard error of the mean (SEM). The relatively small SEM between samples allowed for the minimum group size of three animals. For the Western blot, all data was measured and quantified by ImageJ, and analyzed by one-way analysis of variance (ANOVA). The *post hoc* analysis was conducted with the Tukey’s multiple comparisons unless it is stated otherwise. For sex difference, two-way ANOVA was used to test for differences in means for sex effects and interaction (sex × drug) effects, and Tukey’s multiple comparisons were used to compare each group when significant differences were found. All data was evaluated using GraphPad Prism 7 (GraphPad Software, La Jolla, CA, USA).

## Results

### The Use of Different General Anesthesia and Euthanasia Methods Has a Strong Impact on Extracellular Signal-Regulated Kinase 1 and 2 (ERK1/2) Activity

**Figure 1:**
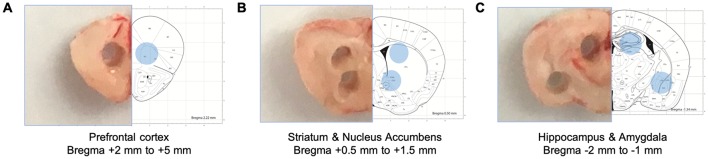
Schematic diagrams of collected brain tissues. Specific brain regions were isolated from brain tissue sections using disposable punches. **(A)** The prefrontal cortex (Bregma +2 mm to +5 mm). **(B)** The dorsal striatum and the nucleus accumbens (Bregma + 0.5 mm to +1.5 mm). **(C)** The dorsal hippocampus (Bregma −2 mm to −1 mm).

**Figure 2:**
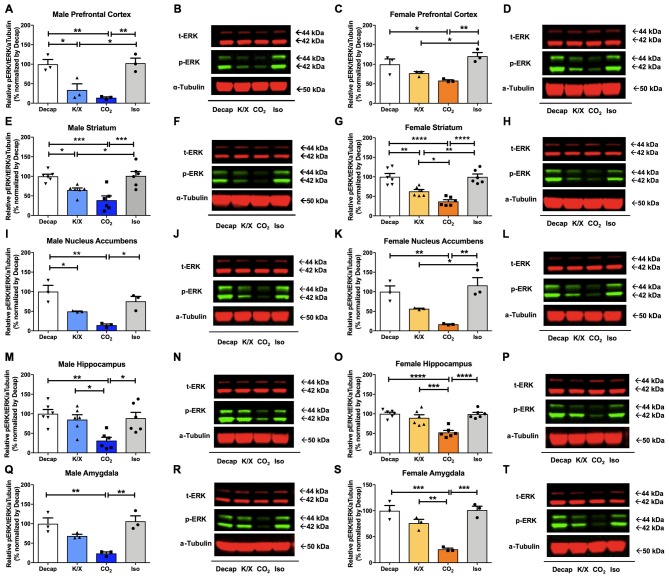
Extracellular signal-regulated kinase 1 and 2 (ERK1/2) activity in male and female brain regions is differentially impacted by anesthesia/euthanasia methods. To test the impact of different anesthetic/euthanasia in mitogen-activated protein kinases (MAPK) activity, ERK1/2 actvity was analyzed by Western blot following the different anesthetic/euthanasia procedures in the male prefrontal cortex **(A,B)**, the male dorsal striatum **(E,F)**, the male nucleus accumbens **(I,J)**, the male dorsal hippocampus **(M,N)**, the male amygdala **(Q,R)**, as well as in the female prefrontal cortex **(C,D)**, the female dorsal striatum **(G,H)**, the female nucleus accumbens **(K,L)**, the female dorsal hippocampus **(O,P)**, and the female amygdala **(S,T)**. Representative Western blot images were presented next to corresponding bar graphs. Each group has three replicates for statistical analysis. All data was represented as mean ± standard error of the mean (SEM; *n* = 3, but *n* = 6 for the male and female dorsal striatum and dorsal hippocampus), and analyzed with one-way analysis of variance (ANOVA) with Tukey’s multiple comparison (*p < 0.05, **p < 0.01, ***p < 0.001, ****p < 0.0001). For abbreviation, “iso” represents isoflurane; “CO_2_” represents carbon dioxide asphyxiation; “K/X” represents ketamine and xylazine administration; “Decap” represents decapitation without anesthesia.

Five different brain regions including the prefrontal cortex, the dorsal striatum, the nucleus accumbens, the amygdala, and the dorsal hippocampus were collected by tissue puncture and further analyzed using Western blot (Figure 1). In all five-brain regions of male mice, one-way ANOVA analysis revealed statistically significant differences in ERK1/2 phosphorylation between euthanasia methods (Figures 2A–R; prefrontal cortex: *F*_(3,8)_ = 14.47, *p* = 0.001; dorsal striatum: *F*_(3,20)_ = 11.74, *p* = 0.0001; nucleus accumbens: *F*_(3,8)_ = 12.88, *p* = 0.002; dorsal hippocampus: *F*_(3,20)_ = 6.63, *p* = 0.003; amygdala: *F*_(3,8)_ = 12.62, *p* = 0.002). Similar results were obtained in the female brain (Figures 2C–T; prefrontal cortex: *F*_(3,8)_ = 9.923, *p* = 0.005; dorsal striatum: *F*_(3,20)_ = 22.56, *p* < 0.0003; nucleus accumbens: *F*_(3,8)_ = 13.04, *p* = 0.002; dorsal hippocampus: *F*_(3,20)_ = 16.18, *p* = 0.02; amygdala: *F*_(3,8)_ = 23.03, *p* = 0.0003). Especially decapitation and isoflurane led to statistically significant increases in ERK1/2 phosphorylation relative to carbon dioxide asphyxiation in both males and females (Figures 2A–R for males; Figures 2C–T for females; see Supplementary Table S1 for *post hoc* multiple comparison). Mice euthanized with ketamine/xylazine displayed stronger ERK1/2 activity than carbon dioxide asphyxiation in the male dorsal hippocampus (Figures 2M,N), the female dorsal striatum (Figures 2G,H), the female dorsal hippocampus (Figures 2O,P), and the female amygdala (Figures 2S,T). Mice euthanized with isoflurane showed more pronounced ERK1/2 activity than ketamine/xylazine in the male prefrontal cortex (Figures 2A,B), the male dorsal striatum (Figures 2E,F), the female prefrontal cortex (Figures 2C,D), the female dorsal striatum (Figures 2G,H), and the female nucleus accumbens (Figures 2K,L; see Supplementary Table S1 for *post hoc* multiple comparison). To increase the reproducibility and scientific rigor of the study, we performed two separate studies using the same paradigm in selected brain regions such as the male and female dorsal striatum and dorsal hippocampus (Supplementary Figure S1). In both trials, we observed similar trends of ERK1/2 activation in general (Supplementary Figure S1; see Supplementary Table S5 for *post hoc* multiple comparison), thus supporting the reproducibility of our results.

### Impact of Different General Anesthesia and Euthanasia Methods on c-Jun N-Terminal Kinase Activity

**Figure 3:**
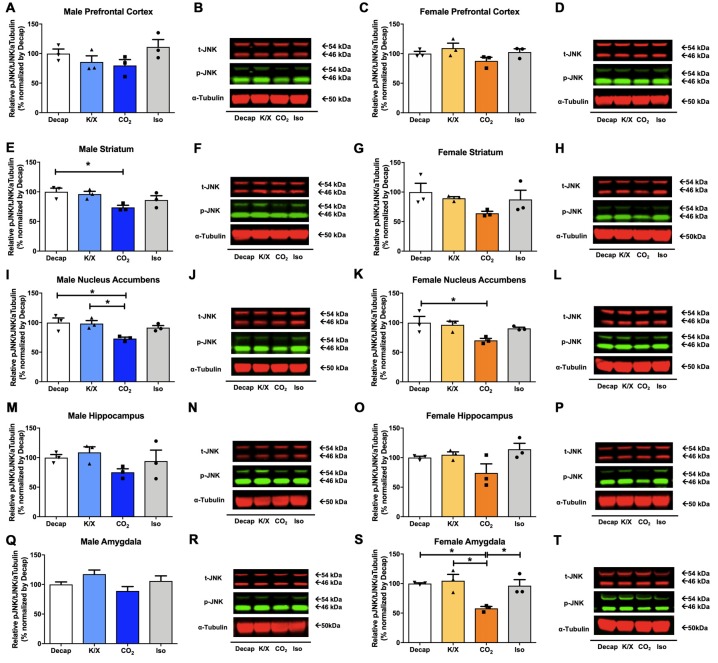
c-Jun N-terminal kinase (JNK) activity in male and female brain regions in response to different anesthesia/euthanasia methods. JNK activity was analyzed by Western blot following the different anesthetic/euthanasia procedures in the male prefrontal cortex **(A,B)**, the male dorsal striatum **(E,F)**, the male nucleus accumbens **(I,J)**, the male dorsal hippocampus **(M,N)**, the male amygdala **(Q,R)**, as well as in the female prefrontal cortex **(C,D)**, the female dorsal striatum **(G,H)**, the female nucleus accumbens **(K,L)**, the female dorsal hippocampus **(O,P)**, and the female amygdala **(S,T)**. Each group has three replicates for statistical analysis. Representative western blot images were presented next to corresponding bar graphs. All data was represented as mean ± SEM, and analyzed with one-way ANOVA with Tukey’s multiple comparison (*p < 0.05).

Of the five tested brain regions, statistically significant differences in JNK phosphorylation between euthanasia methods were only observed in the male dorsal striatum (Figures 3E,F) one-way ANOVA (*F*_(3,8)_ = 4.399, *p* = 0.04), the male nucleus accumbens (Figures 3I,J; *F*_(3,8)_ = 5.765, *p* = 0.02), the female nucleus accumbens (Figures 3K,L; *F*_(3,8)_ = 4.345, *p* = 0.04), the female amygdala (Figures 3S,T; *F*_(3,8)_ = 7.982, *p* = 0.009). However, for the other brain regions we did not find any statistical significant differences between euthanasia methods in males (Figures 3A,B for the prefrontal cortex, *F*_(3,8)_ = 1.898, *p* = 0.2; Figures 3M,N for the dorsal hippocampus, *F*_(3,8)_ = 1.641, *p* = 0.26; Figures 3Q,R for the amygdala, *F*_(3,8)_ = 2.832, *p* = 0.1) nor in females (Figures 3C,D for the prefrontal cortex, *F*_(3,8)_ = 2.162, *p* = 0.17; Figures 3G,H for the dorsal striatum, *F*_(3,8)_ = 1.906, *p* = 0.2; Figures 3O,P for the dorsal hippocampus). Furthermore, decapitation showed a statistical significant increase in JNK activity compared to carbon dioxide asphyxiation in the male dorsal striatum (Figures 3E,F), the male nucleus accumbens (Figures 3I,J), the female nucleus accumbens (Figures 3K,L), and the female amygdala (Figures 3S,T; see Supplementary Table S2 for *post hoc* multiple comparison). Relative to carbon dioxide asphyxiation, ketamine/xylazine usage also produced a statistical significant increase in JNK levels in the male nucleus accumbens (Figures 3I,J) and the female amygdala (Figures 3S,T; see Supplementary Table S2 for *post hoc* multiple comparison). In the female amygdala, a significant increase in JNK levels in response to isoflurane was observed compared to carbon dioxide asphyxiation (Figures 3S,T; see Supplementary Table S2 for *post hoc* multiple comparison).

### Different General Anesthesia and Euthanasia Methods Do Not Impact p38 Kinase Activity

**Figure 4:**
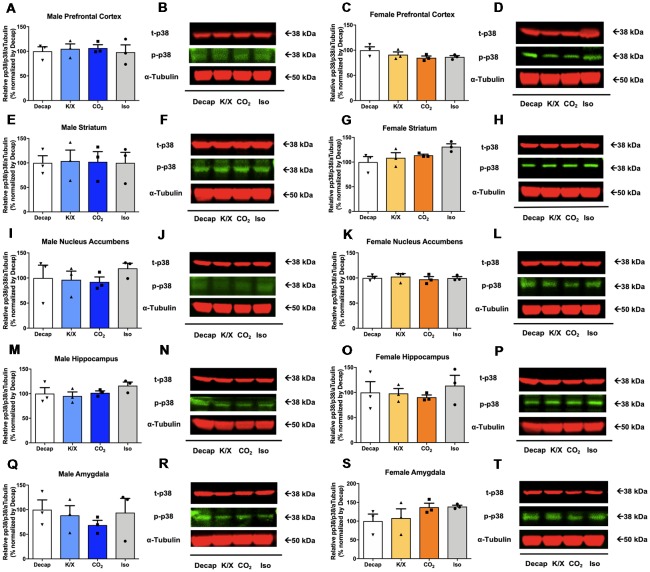
p38 activity in male and female brain regions following different anesthesia/euthanasia methods. p38 activity was analyzed by Western blot following the different anesthetic/euthanasia procedures in the male prefrontal cortex **(A,B)**, the male dorsal striatum **(E,F)**, the male nucleus accumbens **(I,J)**, the male dorsal hippocampus **(M,N)**, the male amygdala **(Q,R)**, as well as in the female prefrontal cortex **(C,D)**, the female dorsal striatum **(G,H)**, the female nucleus accumbens **(K,L)**, the female dorsal hippocampus **(O,P)**, and the female amygdala **(S,T)**. Each group has three replicates for statistical analysis. Representative western blot images were presented next to corresponding bar graphs. All data was represented as mean ± SEM, and analyzed with one-way ANOVA.

No statistical difference between euthanasia methods were observed in p38 activity in males (Figures 4A,B for the prefrontal cortex, *F*_(3,8)_ = 0.1404, *p* = 0.9; Figures 4E,F for the dorsal striatum, *F*_(3,8)_ = 0.008209, *p* = 1.0; Figures 4I,J for the nucleus accumbens, *F*_(3,8)_ = 0.4954, *p* = 0.7; Figures 4M,N for the dorsal hippocampus, *F*_(3,8)_ = 1.146, *p* = 0.4; Figures 4Q,R for the amygdala, *F*_(3,8)_ = 0.429, *p* = 0.7; see Supplementary Table S3 for *post hoc* multiple comparison) or in females (Figures 4C,D for the prefrontal cortex, *F*_(3,8)_ = 1.91, *p* = 0.2; Figures 4G,H for the dorsal striatum, *F*_(3,8)_ = 2.528, *p* = 0.13; Figures 4K,L for the nucleus accumbens, *F*_(3,8)_ = 0.2177, *p* = 0.9; Figures 4O,P for the dorsal hippocampus, *F*_(3,8)_ = 0.37, *p* = 0.78; Figures 4S,T for the amygdala, *F*_(3,8)_ = 1.451, *p* = 0.3; see Supplementary Table S3 for *post hoc* multiple comparison).

### The Effect of Euthanasia Method on ERK1/2 Activity Levels in the Dorsal Hippocampus and the Dorsal Striatum Is Equal for Male and Female Mice

**Figure 5:**
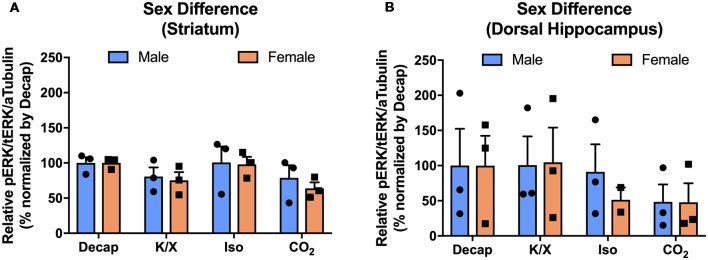
No sex difference of ERK1/2 activation in response to different anesthesia/euthanasia methods in selected brain regions. MAPK activity were normalized to those observed in the decapitation group, and relative levels were analyzed using two-way ANOVA in the stratum **(A)**, the dorsal striatum **(B)**, the dorsal hippocampus for ERK1/2 activity. Representative western blot images were presented in Figure 2. All data was represented as mean ± SEM, and analyzed with two-way ANOVA with Tukey’s multiple comparison.

To investigate if the four euthanasia methods impacted MAPK activity differently between sexes, we tested ERK1/2 activity in the dorsal hippocampus and the dorsal striatum of male and female mice. Using two-way ANOVA, we observed no significant sex effects for ERK1/2 activity between different anesthesia/euthanasia methods in the dorsal striatum (Figure 5A; *F*_(3,16)_ = 0.1196, *p* = 0.9473), and the dorsal hippocampus (Figure 5B; *F*_(3,15)_ = 0.1157, *p* = 9495 see Supplementary Table S4 for *post hoc* multiple comparison).

## Discussion

In the current study we evaluated to what degree four commonly used general anesthesia/euthanasia methods for brain tissue collection affect MAPK activity in the naïve and healthy adult male and female wild-type mice. Of the four methods (isoflurane, carbon dioxide asphyxiation, ketamine/xylazine, and rapid unanesthetized decapitation), those brains collected following carbon dioxide asphyxiation showed the lowest ERK1/2 phosphorylation in the prefrontal cortex, the dorsal striatum, the nucleus accumbens, the dorsal hippocampus, and the amygdala of male and female adult mice. The differences in basal JNK activity between the four methods were less pronounced compared to those observed for ERK1/2 activity. More specifically, except for the male dorsal striatum, the male and female nucleus accumbens, and the female amygdala, no significant differences were observed in JNK activity between the different anesthesia/euthanasia methods. No statistical significant differences between groups were observed in terms of p38 activity. Our findings are in agreement with a previous study investigating the impact of different anesthesia methods on metabolomics of mammalian tissue (Overmyer et al., 2015). This study, which did not study the brain, found that carbon dioxide generally produced few significant changes in metabolomics, whereas isoflurane, ketamine and pentobarbital produced more significant changes. Our study complements this previous study as similar C57BL/6 mice of comparable age were used, although our experiments were performed during the mice’s active cycle (in the dark), and focused on MAPK activity in five brain regions linked to various neuropsychiatric disorders.

Although there are many brain regions that are involved in the modulation of MAPK signaling pathways, we specifically chose the prefrontal cortex, the dorsal hippocampus, the amygdala, the nucleus accumbens, and the dorsal striatum as these brain regions are some of the most commonly investigated in the field of neuroscience particular in relation to neuropsychiatric disorders. For example, the hippocampus and the amygdala are important brain regions for anxiety disorder (Bannerman et al., 2004), memory disorder (Bannerman et al., 2004), and may also be involved in attention-deficit and hyperactivity disorder (Onnink et al., 2014), and autistic spectrum disorder (Tottenham et al., 2014). For example, consolidation and extinction fear memory required ERK1/2 in the hippocampus and the basolateral amygdala (Besnard et al., 2014). The dorsal striatum and the nucleus accumbens are heavily implicated in substance use disorder (Robbins and Everitt, 2002), Parkinson’s disease (Surmeier et al., 2014), and epilepsy (Deransart et al., 2000). Especially MAPK signaling pathway in striatal neurons involved in the formation of neuronal plasticity related to addictive behavior (Wang et al., 2004). Furthermore, abnormalities in the prefrontal cortex are also implicated in attention-deficit/hyperactivity disorder (Halperin and Schulz, 2006), autistic spectrum disorder (Morgan et al., 2010; Courchesne et al., 2011), and various mood disorders (Drevets et al., 1997). For instance, MAPK activation mediated by dopaminergic D_1_ and D_2_ receptors in the prefrontal cortex, which are important for various mood and motor control, is also linked to neuronal plasticity (Goto et al., 2010). Therefore, in order to study the neurochemical basis of these disorders or how drug treatments correct or alter the cellular signaling, it is important to properly assess MAPK activity levels. However, as our study reveals, methods commonly used for collecting brain tissue may fundamentally change MAPK activity even in naïve wild-type mice. To ensure we obtained enough tissue for our experiments, we chose to use micropunches rather than laser capture microdissection. A limitation of this method is that besides the targeted brain region, we also recovered some adjacent tissue. It is possible that MAPK activity in these nearby regions contributed or even dominated the effect we observed. Still, that would not negate the patterns we observe when comparing the different euthanasia methods as those are relative.

In our study, we demonstrated that four anesthetic/euthanasia methods differentially impact three MAPKs. We used decapitation as a non-chemical-assisted method as our control group. The choice of euthanasia method had the largest impact on ERK1/2 activity levels. Relative to carbon dioxide asphyxiation, isoflurane produced strong ERK1/2 activation in all brain regions of both male and female mice. It has been reported that a low dose (0.7%) of isoflurane for a short-term (30 min) exposure increased N-Methyl-D-aspartate (NMDA) receptor subtype 2B (NR2B) and its downstream ERK1/2 phosphorylation, but a high dose (1.4%) of isoflurane with a long-term (4 h) exposure decreased NR2B and ERK1/2 activity as well as increased neuroapoptosis in the mouse hippocampus (Liu et al., 2014). In this study, we briefly (5 min) induced with a 5% isoflurane dose, which, compared to the Liu et al experiments, may more closely mimic the 30 min 0.7% exposure; the condition that increased NR2B and ERK1/2 activity.

We also observed that decapitation increased ERK1/2 activity relative to carbon dioxide asphyxiation in all tested brain regions of male and female adult mice. These findings appear to be in line with previous reports on stress hormone-induced ERK1/2 activation. Acute stress may play a role in the modulation of stress hormone receptors such as corticotropin releasing hormone (CRH) receptors and glucocorticoid receptors (Meller et al., 2003). Previous studies have shown ERK1/2 activation by acute stress-mediated modulation of stress-related receptors such as CRH receptors and glucocorticoid receptors (Meller et al., 2003; Kim et al., 2018), suggesting a potential implication of stress in ERK1/2 activation.

Systemic administration of ketamine/xylazine also led to significant ERK1/2 activation in the male dorsal striatum, the male dorsal hippocampus, and the female amygdala compared to carbon dioxide asphyxiation. It is possible that ketamine activated the mammalian target of rapamycin pathway as a NMDA receptor antagonist, increasing ERK1/2 phosphorylation in the brain (Li et al., 2010).

JNK activation is reportedly involved in the isoflurane-mediated neuroapoptosis of the hippocampus of neonatal rats (Li et al., 2013; Liao et al., 2014). Yet, we only observed that isoflurane increased JNK activation relative to carbon dioxide asphyxiation in the amygdala of female mice. As the previous studies used neonatal rats, it is possible that the differences in age and species between our and their studies underlie the differences in outcome. Additionally, it is also possible that the duration and percentage of isoflurane were not comparable to observe similar trends in JNK activation in our hippocampal sections.

For p38 activity, we originally hypothesized that carbon dioxide asphyxiation similar to a reduced oxygen condition (hypoxic condition) may increase p38 activity, because a deprivation of oxygen supply by ischemia/hypoxia-induced cerebral injury in the brain may induce activation of p38 leading to neuroapoptosis (Bu et al., 2007; Yang et al., 2013). However, we did not observe any changes in p38 activity. This could be partly due to the low intensity of the p38 and p-p38 signal compared to ERK1/2 or JNK. This may either be due to actual low presence of p38 in these regions or relative poor quality of the p38/p-p38 antibodies. Another possibility is that more prolonged exposure to low oxygen levels or other types of cellular injury may be required to induce measurable p38 activation (Hensley et al., 2000).

In our study, we did not measure ERK activity in alive animals using biosensors, instead we relied on euthanasia to collect brains. As such this study does not contain a negative control or sham condition and therefore it is unclear what baseline MAPK activity truly is. Thus, we are unable to claim if isoflurane increased ERK1/2 phosphorylation or carbon dioxide asphyxiation decreased ERK1/2 activity. However, because there are mechanistic rationales for how isoflurane and ketamine can induce activation of MAPK pathways (Réus et al., 2014), the most logical hypothesis is that the observed MAPK levels obtained from carbon dioxide exposed mice are closest to the actual baseline of MAPK activity in adult mice brains.

We also explored sex as an independent variable. While a previous study found that sex hormones impacts MAPK activity through a mechanism involving Tropomyosin-Related Kinase B (TrkB) receptors (Carbone and Handa, 2013), we did not observe sex effects in selected brain regions such as the dorsal hippocampus and the dorsal striatum.

One limitation of our study is that we only used a single mouse strain, specifically the C57BL/6 strain, but given that the C57BL/6 strain is one of the most commonly employed strains in various neurological studies, the findings should still be broadly informative for researchers aiming to optimize brain tissue collections for investigation of MAPK activity. Another potential limitation is that we used only healthy and drug-naïve mice. Mice suffering from specific neurological conditions or undergoing drug treatments may become more or less susceptible to one or more of the different anesthesia/euthanasia methods, and thus caution is advised when extrapolating our results to diseased or drug-treated animals. Yet, we are encouraged by the similarity of our finding compared to the metabolomics study of these methods (Overmyer et al., 2015). Another limitation of our study is that we did not perfuse the brains with saline to remove blood, as lymphocytes in the blood can contribute to the production of cytokines and activate MAPK signaling through tyrosine kinase (Grace et al., 2014). However, we did not perfuse any of the collected brains, and thus the observed differential MAPK activity levels following the four euthanasia methods cannot be attributed to white blood cell induced MAPK activity.

## Conclusion

Our results demonstrated that euthanasia by carbon dioxide asphyxiation prior to brain tissue collection provides minimal naïve MAPK activation. Based on our findings, we recommend the usage of carbon dioxide asphyxiation as the method of choice for terminal euthanasia for brain tissue collection. Furthermore, for procedures such as transcardiac perfusion for immunohistochemistry or stereotaxic surgery that require a prolonged anesthetic method, we would recommend a systemic administration of ketamine/xylazine over the use of isoflurane. Still, we do recommend careful consideration of the selection of dosages and time-points of euthanasia following anesthesia as these may impact the degree of MAPK activity. The presented findings offer important information for researchers who are seeking methods to optimize brain collection to investigate MAPK activity in ways that reduce the risk of false negative results.

## Author Contributions

RR supervised MK and GM. RR and MK designed the experiments. MK and GM carried out the experiments. MK, GM and RR analyzed the data, edited and proofread the manuscript and approved the final draft. MK wrote the first draft of the manuscript.

## Conflict of Interest Statement

The authors declare that the research was conducted in the absence of any commercial or financial relationships that could be construed as a potential conflict of interest.
