# Supplementary material for: Commonly Used Anesthesia/Euthanasia Methods for Brain Collection Differentially Impact MAPK Activity in Male and Female C57BL/6 Mice
Source: Front Cell Neurosci. 2019 Mar 28;13:96. doi: 10.3389/fncel.2019.00096 (PMC6447702; doi:10.3389/fncel.2019.00096)
Supplement: Supplementary file 1 [file Image_1.pdf]

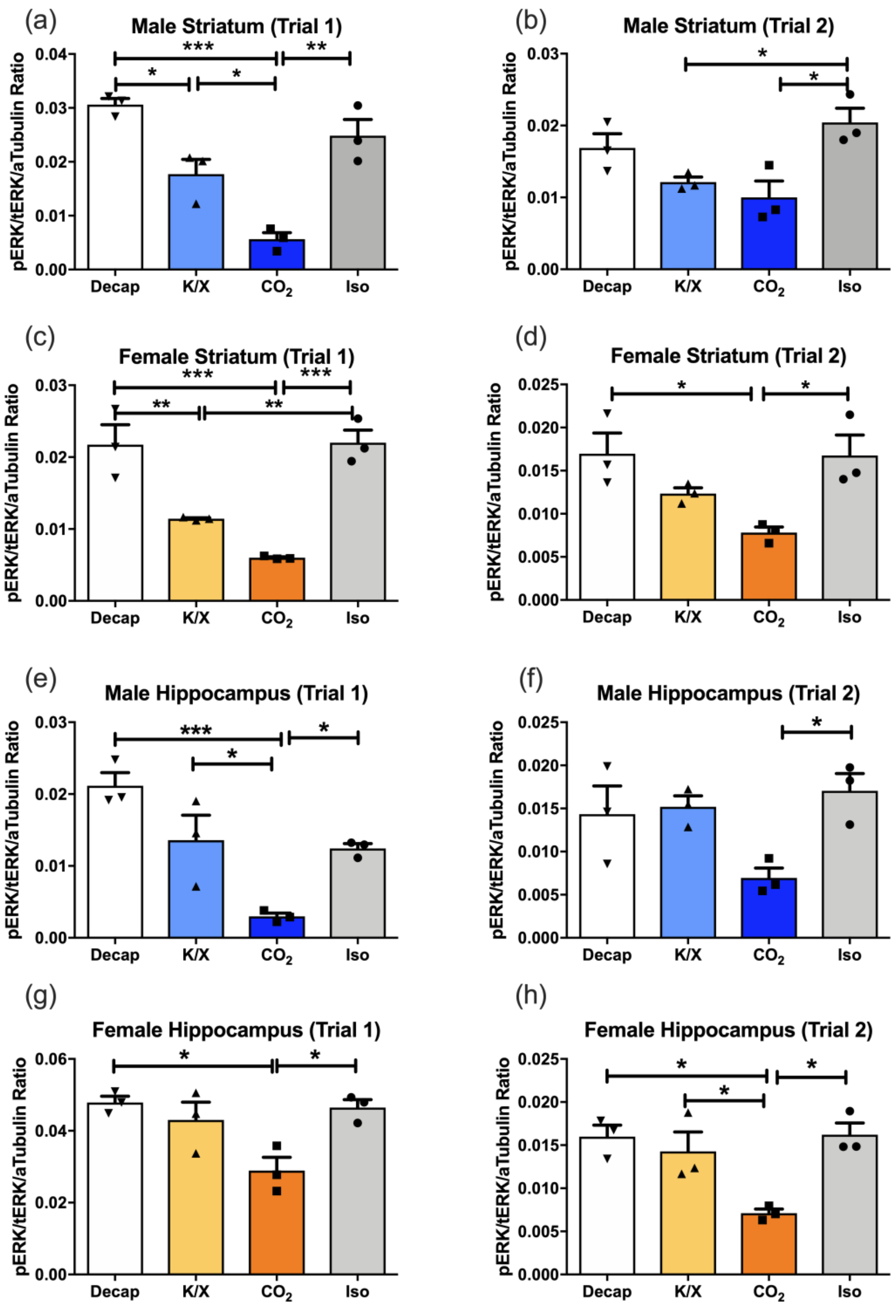

Supplementary Figure S1: Reproducibility of the experimental paradigm was tested in selected brain regions using the same four anesthetic and euthanasia methods. To increase the scientific rigor and reproducibility, ERK1/2 activation in the male dorsal striatum (A,B), the female dorsal striatum (C,D), the male dorsal hippocampus (E,F), and the female dorsal hippocampus (G,H) to different euthanasia methods were tested in two separate trials.
